# Determinants of tobacco use among pregnant women in sub-Saharan Africa. A multilevel mixed-effect logistic regression model

**DOI:** 10.1371/journal.pone.0297021

**Published:** 2024-05-21

**Authors:** Setognal Birara Aychiluhm, Kusse Urmale Mare, Betelhem Dagnew, Abubeker Alebachew Seid, Mequannent Sharew Melaku, Kebede Gemeda Sabo, Abay Woday Tadesse, Kedir Y. Ahmed

**Affiliations:** 1 Department of Epidemiology and Biostatistics, Institute of Public Health, College of Medicine and Health Sciences, University of Gondar, Gondar, Ethiopia; 2 Department of Nursing, College of Medicine and Health Sciences, Samara University, Samara, Ethiopia; 3 College of Veterinary Medicine, Samara University, Samara, Ethiopia; 4 Department of Health Informatics, Institute of Public Health, University of Gondar, Gondar, Ethiopia; 5 Curtin School of Population Health, Curtin University, Bentley, Western Australia, Australia; 6 Rural Health Research Institute, Charles Sturt University, Orange, New South Wales, Australia; 7 Translational Health Research Institute, Western Sydney University, Campbelltown, New South Wales, Australia; Eduardo Mondlane University: Universidade Eduardo Mondlane, MOZAMBIQUE

## Abstract

**Introduction:**

Although it is known that maternal tobacco use during pregnancy substantially declined in higher-income countries, information on the magnitude and determinants of tobacco use among pregnant women in sub-Saharan Africa (SSA) remains limited. Establishing evidence on maternal tobacco during pregnancy is crucial for guiding targeted interventions in SSA. This study aimed to determine the overall prevalence of tobacco use and its determinants among pregnant women in SSA countries.

**Methods:**

The study used data from Demographic and Health Surveys conducted in 33 countries across SSA from 2010 and 2021. Our analysis included a total weighted sample of 40,291 pregnant women. A multilevel logistic regression model was used to identify factors associated with maternal tobacco use during pregnancy. The measure of association between explanatory variables and the outcome was reported using adjusted odds ratios (AORs) with 95% confidence intervals (CIs).

**Results:**

The pooled prevalence of tobacco use among pregnant women in SSA was 1.76% (95% CI: 1.41, 2.12). Our findings showed that pregnant women in the age groups of 25–34 years (AOR 1.44; 95% CI: 1.14, 1.82) and 35+ years (AOR 2.18; 95% CI: 1.68, 2.83) had higher odds of tobacco use during pregnancy. Pregnant women who attained primary education (AOR 0.57; 95% CI: 0.46, 0.70) and secondary or higher education (AOR 0.39; 95% CI: 0.30, 0.53) were associated with lower odds of tobacco use. Similarly, pregnant women who resided in households with a high wealth index (AOR 0.36; 95% CI: 0.55 0.90) and those with media exposure (AOR 0.81; 95% CI: 0.67, 0.99) were less likely to use tobacco during pregnancy.

**Conclusion:**

This study revealed that the overall prevalence of maternal tobacco use during pregnancy was relatively low in SSA, but some countries exhibited higher estimates. To address this, it is crucial to implement targeted smoking prevention and cessation strategies, particularly for young pregnant women, those facing socioeconomic disadvantages, and those with lower educational status.

## Introduction

Globally, tobacco smoking is a major public health concern, causing over seven million deaths annually, with six million attributed to active smoking and the remainder to passive exposure [1]. Given these statistics, several global initiatives have been implemented to decrease the use of tobacco products [2]. In 2003, the World Health Assembly adopted the World Health Organization (WHO) Framework Convention on Tobacco Control (FCTC) in response to the globalization of the tobacco epidemic [3]. As part of these efforts, the WHO has also set a 30% decrease in tobacco usage as a global objective by 2025 [4]. Emphasizing the importance of addressing tobacco use during pregnancy is crucial for ensuring the health and well-being of both mothers and their infants [5].

Maternal exposure to tobacco during pregnancy has both short-term and long-term consequences [6]. In the short term, exposure to tobacco products during pregnancy leads to complications such as placenta previa, placental abruption, and pre-eclampsia, along with adverse fetal outcomes including low birth weight, premature birth, and complete perinatal death [7,8]. Additionally, first-trimester maternal smoking is associated with congenital anomalies, including atrial septal anomalies, transposition of the main arteries, conotruncal malformations, and patent ductus arteriosus [9].

In the long term, maternal smoking during pregnancy is known to have adverse effects on children, including attention deficit disorders and learning disabilities, childhood cancer and obesity, elevated blood pressure in children, and a lack of breast milk due to insufficient lactation [10–12]. Smokeless tobacco usage among pregnant women has also consistently shown strong associations with important factors such as stress or depression, alcohol-drinking behaviors, and low educational attainment [13].

In 2016, the prevalence of smoking during pregnancy ranged from 1.7% to 4.5%, with variations from 0.8% in the African region to 8.1% in the European region [14]. While it is known that maternal tobacco use during pregnancy substantially declined in higher-income countries [15], information on the overall magnitude and determinants of tobacco use among pregnant women in sub-Saharan Africa (SSA) remains limited. The prevalence of tobacco use in SSA countries was expected to undergo the largest increase [16] as the SSA region is still in the early stage of the tobacco epidemic [17].

Previously published studies have shown that the partner’s smoking status, partner’s educational status, occupation, residence site, marital status, number of previous pregnancies, and adequate prenatal care are significant risk factors associated with tobacco use during pregnancy [18–22]. Understanding the overall prevalence and determinants of tobacco use among pregnant mothers is essential for guiding policy interventions in SSA. Therefore, this study aimed to determine the pooled prevalence of tobacco use and its determinants in 33 sub-Saharan African countries.

## Methods

### Data source and participants

This study used the latest available Demographic and Health Surveys (DHS) data sets from 33 SSA countries conducted between 2010 and 2021. Out of the 44 SSA countries with routine DHS, five countries (Central African Republic, Sudan, Nigeria Ondo State, Eswatini, and Sao Tome and Principe) had data older than 2010, four countries (Cape Verde, Equatorial Guinea, Botswana, and Eritrea) had data restrictions, and two countries (Chad and Senegal) had no observation on the outcome variable of tobacco use. In the end, our final analysis included 33 countries, including Angola, Benin, Burkina Faso, Burundi, Comoros, Ethiopia, Kenya, Democratic Republic of Congo, Congo, Cote D’Ivoire, Cameroon, Gabon, Ghana, Gambia, Guinea, Liberia, Lesotho, Madagascar, Mali, Mauritania, Malawi, Mozambique, Nigeria, Niger, Namibia, Rwanda, Sierra Leone, Togo, Tanzania, Uganda, South Africa, Zambia and Zimbabwe.

The DHS surveys across all countries employed standardized procedures to gather data on basic socio-demographic characteristics and different health indicators. In this analysis, since the study population was pregnant women, we extracted women’s records (IR) from each country and then appended them after managing missing observations. Finally, a total weighted sample of 40,206 pregnant women was included in the analysis. The data is available in the public domain and accessed at https://dhsprogram.com/data/available-datasets.cfm.

### Study variables

#### Outcome variable

The main outcome variable for this study was maternal tobacco use during pregnancy. This was measured using pregnant women’s self-reported use of various tobacco products, including cigarettes, pipes, chewing tobacco, snuffs by a nose, snuffs by mouth, smoking kreteks, smoking cigars/cheroots/cigarillos, smoking water pipes, and other country-specific tobacco products. Pregnant women were classified as “Tobacco users” if the response was ‘Yes’ for any of the above tobacco products; otherwise, they were classified as “Non-tobacco users”.

#### Independent variables

We grouped independent variables into individual and community-level variables. Individual-level variables included age, marital status, women’s educational status, women’s current working status, partner’s education level, household wealth index, and media exposure. On the other hand, community-level variables included residence, region, community-level women’s literacy, community-level media exposure, and community-level poverty. Community-level variables, specifically community-level women’s literacy, community-level media exposure, and community-level poverty, were generated by aggregating the individual-level observations at the cluster level. Median values were then used to categorize the generated variables as “low” and “high”.

### Statistical analysis and modeling

All analyses were conducted using STATA software (version 14.1, Stata Corp, College Station, TX, USA). In our analyses, we considered sampling weight to address potential imbalances or unequal probabilities in household selections and non-responses, as well as to consider clustering and stratification. Frequency and percentages were used to present descriptive statistics. To account for the hierarchal nature of DHS data, a multilevel logistic regression model was used to identify determinants of tobacco use among pregnant women.

#### Model building and comparison

The DHS data is structured hierarchically, with women nested within clusters. Pregnant women in each cluster are anticipated to have greater similarities compared to the broader population. This suggests that advanced models need to account for the variability between clusters. To address this, the mixed-effect logistic regression analysis method was employed.

The multi-level models were specified in four steps. In the initial step, model I (null model) was fitted without independent variables to test random variability in the intercept. Subsequently, model II was then fitted with only community-level explanatory variables. In model III, a model with only individual-level explanatory variables was used. Finally, model IV (full model) was used based on both individual and community-level predictors simultaneously. The formula for the fitted model was as follows:

$$\text{log}\left[\frac{\pi ij}{1-\pi ij}\right]+\beta_{0}+\beta_{1}{\text{X}}_{1\text{ij}}+\ldots \beta_{n}{\text{X}}_{\text{nij}}+{\text{u}}_{\text{oj}}+{\text{e}}_{\text{ij}},$$

Where π_ij_ is the probability of pregnant women who use tobacco; 1—π_ij_ is the probability of pregnant women who do not use tobacco; β_0_ is log odds of the intercept β_1_… β_n_ is the amount of effect by the individual and community-level variables; X_1_…X_n_ represent the independent variables at the individual and community level; u_oj_ is the random error at community (cluster); and e_ij_ is the random error at the individual level.

Additionally, the intra-class correlation coefficient (ICC), Median odds ratio (MOR), and Proportional Change in Variance (PCV) were calculated to check the random variation across clusters. The ICC was calculated as the proportion of the between cluster variation in the total variation:

$$ICC=\left[\frac{Var\left(\text{uoj}\right)}{Var\left(Uoj\right)+\pi^{2}/3}\right]$$

Where Var(uoj) is the community (cluster) level variance and Π^2^/3 is the standard logistic distribution which is Π^2^/3 = 3.29 [23]. The MOR is defined as the median value of the odds ratio between the cluster at the highest risk and the cluster at the lowest risk when randomly picking out two clusters. MOR was calculated using the formula

$$MOR=e0.95\sqrt{\text{Var}\left(\text{uoj}\right)}$$

where Var(uoj) is a community (cluster) level variance [23,24]

The variability in the odds of tobacco use among pregnant women can be explained by successive models calculated by PCV using the following formula:

$$PCV=\left[\frac{Ve-Vmi}{Ve}\right],$$

Where Ve is the variance in the null model and Vmi is the variance in the successive models.

The model selection was based on Akaike’s information criterion (AIC), favoring the one with the lowest AIC value. Model IV (full model), a model with both individual and community-level predictors, demonstrated the smallest AIC value. The measure of association between explanatory variables and the outcome was reported using adjusted odds ratios (AORs) with 95% confidence intervals (CIs).

### Ethical statement

We requested authorization to access data from the Measure DHS program and obtained approval (AuthLetter_186779) to download and use the data for our study from the DHS website (http://www.measuredhs.com). The requirement for informed consent was waived as secondary data were used.

## Results

### Sociodemographic characteristics

The study included a weighted total sample of 40,206 pregnant women. Among these, 27,988 (69.61%) were married, and 12,632 (31.42%) had completed secondary education or higher. A total of 26,665 (66.32%) pregnant women resided in rural areas, and 17,323(42.99%) were from the Western African region. Our findings also found that 17,426 (43.34%) pregnant women resided in households with a low wealth index (Table 1).

**Table 1:** Prevalence of tobacco use across individual and community-level characteristics of pregnant women in SSA region.

		Use of Tobacco		
Variable	Category	Yes	No	Frequency (%)
Age category	≤24 years	226 (29.56)	16,340 (41.43)	16,566 (41.20)
	25–34 years	344 (44.96)	17,193 (43.59)	17,537 (43.62)
	≥35 years	195 (25.48)	5,907 (14.98)	6,103 (15.18)
Residence	Urban	176 (22.98)	13,366 (33.89)	13,541 (33.68)
	Rural	590 (77.02)	26,075 (66.11)	26,665 (66.32)
Wealth Index	Low wealth index	437(57.02)	16,989 (43.08)	17,426 (43.34)
	Middle wealth index	137 (17.91)	7,913 (20.06)	8,050 (20.02)
	High wealth index	192 (25.07)	14,538 (36.86)	14,730 (36.64)
Marital Status	Married	528 (68.92)	27,460 (69.62)	27,988 (69.61)
	Living with partner	165 (21.54)	7,413 (18.80)	7,578 (18.85)
	Others*	73 (9.54)	4,567 (11.58)	4,640 (11.54)
Women education level	No formal education	380 (49.68)	13,685 (34.70)	14,066 (34.99)
	Primary education	256 (33.45)	13,252 (33.60)	13,508 (33.60)
	Secondary or higher education	129 (16.88)	12,502 (31.70)	12,632 (31.42)
Husband education level	No formal education	327 (45.95)	12,070 (35.10)	12,398 (35.32)
	Primary education	221 (31.01)	9,502 (27.63)	9,723 (27.70)
	Secondary or higher education	164 (23.04)	12,816 (37.27)	12,980 (36.98)
Current working status of women	Yes	480 (62.67)	22,321 (58.14)	22,801 (58.23)
	No	286 (37.33)	16,072 (41.86)	16,358 (41.77)
Media exposure	No	350 (45.73)	13,144 (33.33)	13,495(33.56)
	Yes	416 (54.27)	26,296 (66.67)	26,712 (66.44)
SSA region	Central Africa	122 (15.91)	6,499 (16.48)	6,987 (17.34)
	Eastern Africa	319 (41.66)	13,281 (33.67)	13,571 (33.68)
	Southern Africa	68 (8.93)	2,481 (6.29)	2,410 (5.98)
	Western Africa	257 (33.50)	17,178 (43.55)	17,323 (42.99)
Community media exposure	Low	354 (46.27)	20,103 (50.97)	20,458 (50.88)
	High	412 (53.73)	19,337 (49.03)	19,749 (49.12)
Community-women education	Low	365 (47.68)	20,084 (50.92)	20,449 (50.86)
	High	20,449(50.86)	19,357 (49.08)	19,757 (49.14)
Community poverty level	Low	383 (49.95)	20,401 (51.73)	20,784 (51.69)
	High	383 (50.05)	19,039 (48.27)	19,423 (48.31)

### Prevalence of tobacco product use among pregnant women in SSA

**Fig 1:**
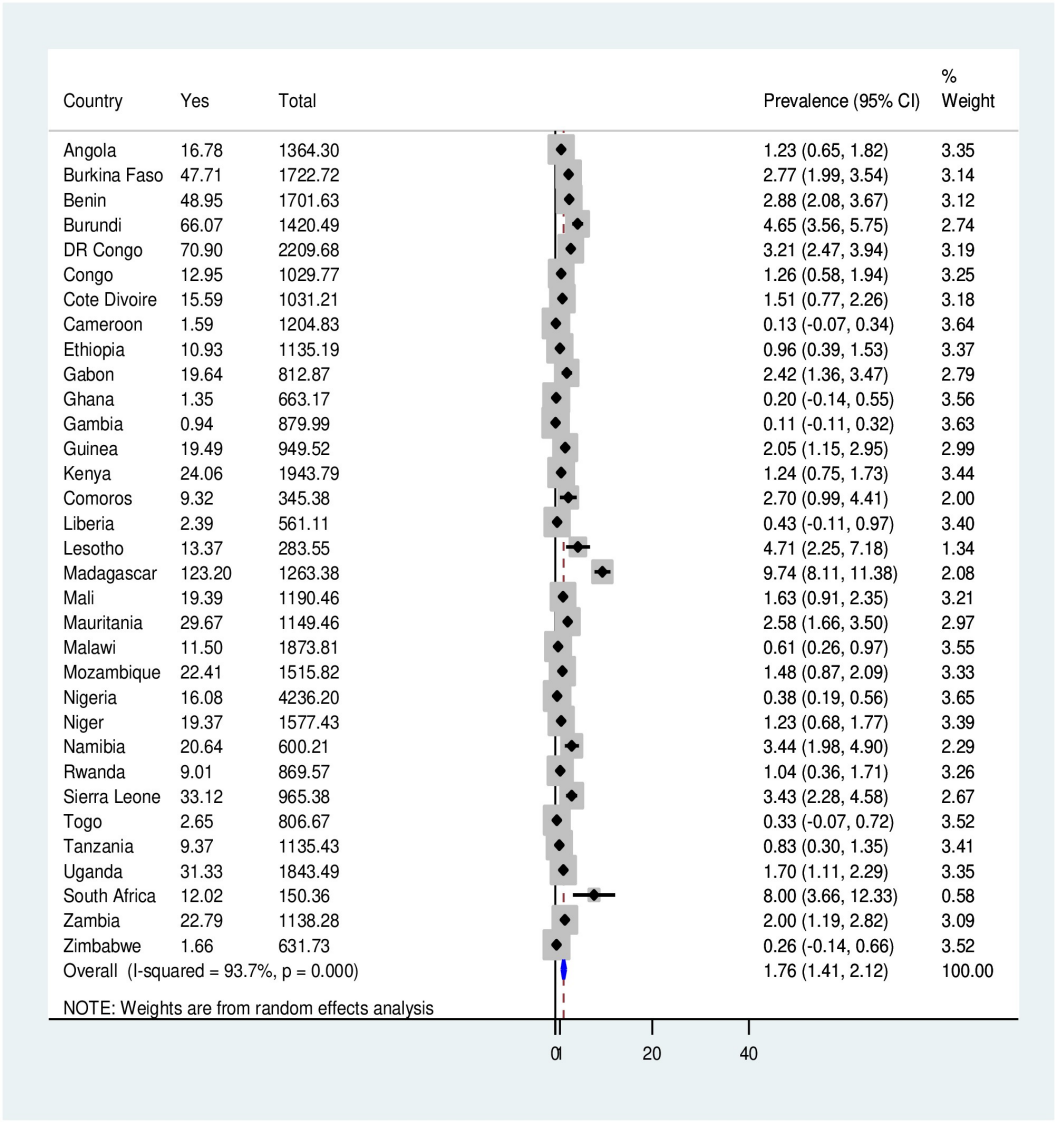
Forest plot showing the pooled prevalence of tobacco use among pregnant women in 33 SSA countries. Further subgroup analyses were conducted considering the higher heterogeneity (higher I^2^ value) in the prevalence of tobacco use among pregnant women across SSA countries. The results from the subgroup analysis showed that the pooled prevalence of tobacco use among pregnant women ranged from 1.38% (95% CI: 0.91, 1.84) in the West African Region to 2.65% (95% CI: 1.13, 4.17) in the South African Region (Fig 2).

The pooled prevalence of tobacco product use among pregnant women in SSA was 1.76% (95% CI: 1.41, 2.12). Madagascar had the highest prevalence of tobacco use during pregnancy (Prevalence = 9.74%; 95% CI: 8.11, 11.38), followed by South Africa (Prevalence = 8.00%; 95% CI: 3.66, 12.33) and Lesotho (Prevalence = 4.71%; 95% CI: 2.25, 7.18). The lowest prevalence of tobacco use during pregnancy was found inGambia (Prevalence = 0.11%; 95% CI:-0.11, 0.32), followed by Cameroon (Prevalence = 0.13%; 95% CI:-0.07, 0.34) (Fig 1).

**Fig 2:**
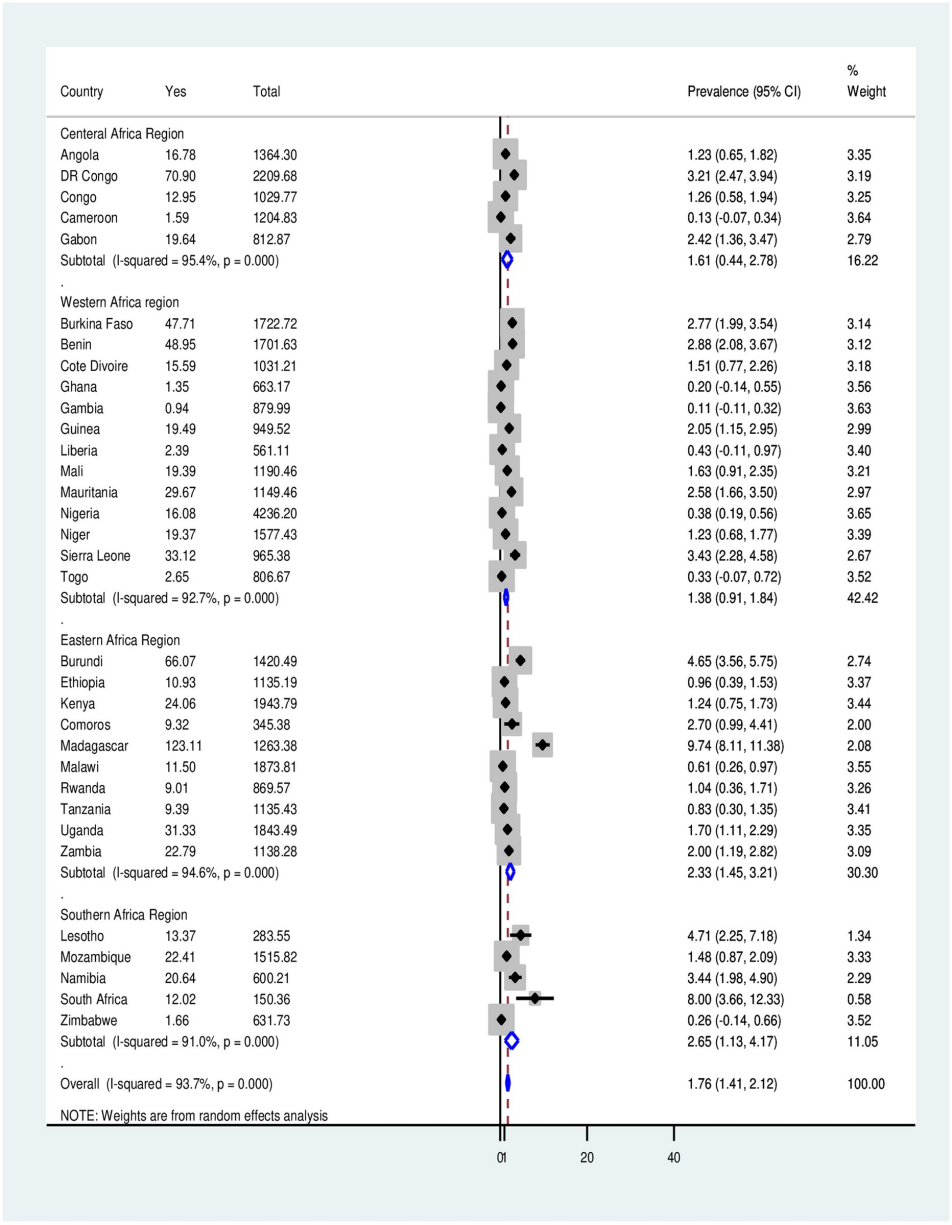
Sub-group analysis of tobacco product use among pregnant women across SSA Regions.

### Random effect analysis

The ICC value in the null model showed a 14.5% variation in tobacco product use among pregnant women across clusters. The PCV value for the final model showed that the combined effect of individual and community-level variables explains 44.6% of the variance in tobacco use at the community level. Furthermore, the presence of heterogeneity in tobacco use levels across clusters was indicated by the MOR, with a value of 1.93 in the null model. This implies that the likelihood of tobacco use among pregnant women in clusters with higher tobacco use prevalence was nearly twofold compared to those in clusters with a lower tobacco use level. Model IV had the lowest AIC value (i.e. 6867), indicating the best-fitted model (Table 2).

**Table 2:** The result of random-effect logit models in predicting tobacco use among pregnant women in 33 SSA countries.

Parameters	Null Model	Model-II	Mode-III	Model-IV
Variance	0.56	0.54	0.49	0.31
Intraclass correlation coefficient	14.5	14	12.9	0.09
Proportion change in variance (in %)	Reference	3.6	12.5	44.6
Median odds ratio	1.93	1.90	1.80	1.44
**Model fitness**				
AIC	7127	6874	7108	6867
BIC	7144	7011	7160	6970

### Determinants of tobacco product use among pregnant women in SSA

In the multilevel multivariable logistic regression model, our findings showed that pregnant women aged 25–34 years and 35+ years were 1.44 times (AOR 1.44; 95% CI: 1.14, 1.82) and 2.18 times (AOR 2.18; 95% CI: 1.68, 2.83) more likely, respectively, to use tobacco products during pregnancy compared to women aged 24 years or younger, after adjusting for other covariates.

Pregnant women who had completed primary schooling were 43% less likely to use tobacco products (AOR 0.57; 95% CI: 0.46, 0.70) and those who had completed secondary education or higher were 61% less likely (AOR 0.39; 95% CI: 0.30, 0.53) compared to pregnant women who had no formal education. The odds of tobacco use during pregnancy among pregnant women with media exposure was lower by 19% compared to women who had no media exposure (AOR 0.81; 95% CI: 0.67, 0.99). Furthermore, pregnant women who resided in households with a high wealth index had 64% lower odds of tobacco product use compared to those pregnant women from households with a low wealth index (AOR 0.36; 95% CI: 0.55 0.90) (Table 3).

**Table 3:** Multilevel multivariable logistic regression for determinants of tobacco use among pregnant women in 33 SSA countries.

		AOR(95%CI)		
Variable	Category	Model II	Model III	Model V
Age category	≤24 years	-	-	-
	25–34 years	-	1.46 (1.13–1.88)	1.44 (1.15–1.82)*
	≥35 years	-	2.29 (1.64–3.22)	2.18 (1.68–2.83)*
Wealth Index	Low wealth index	-	-	-
	Middle wealth index	-	0.73 (0.58–0.94)	0.77 (0.59–1.01)
	High wealth index	-	0.67 (0.47–0.96)	0.70 (0.55–0.90)*
Marital Status	Married	-	-	-
	Living with partner	-	1.20 (0.85–1.70)	1.20 (0.95–1.52)
	Others*	-	0.90 (0.55–1.47)	1.01 (0.75–1.36)
Women education level	No formal education	-	-	-
	Primary education	-	0.61 (0.47–0.80)	0.57 (0.46–0.70)*
	Secondary or higher education	-	0.37 (0.27–0.51)	0.39 (0.30–0.53)*
Current working status of women	No	-	-	-
	Yes	-	0.88 (0.67–1.16)	1.12 (0.91–1.37)
Media exposure	No	-	-	-
	Yes	-	0.90 (0.72–1.13)	0.81 (0.67–0.99)*
Residence	Urban	-	-	-
	Rural	1.65 (1.31–2.08)	-	0.98 (0.76–1.26)
Community media exposure	Low	-	-	
	High	1.16 (0.95–1.41)	-	1.04 (0.86–1.27)
Community-women education	Low	-	-	
	High	1.13 (0.92–1.38)	-	0.97 (0.79–1.20)
Community poverty level	Low	-	-	
	High	0.91 (0.74–1.10)	-	0.87 (0.71–1.07)

## Discussion

This study has provided evidence on tobacco use among pregnant women using representative data from 33 SSA countries. Our findings are crucial for governments and policy-makers to assess and enhance Tobacco Control Directives, particularly for a special group of populations such as pregnant women. To the best of our knowledge, this is the first study to investigate the overall prevalence of tobacco product use and its determinants among pregnant women in 33 SSA countries using nationally representative data.

Our findings indicated a comparatively low overall prevalence of tobacco product use among pregnant women in 33 SSA countries, standing at 1.8%. This figure is slightly different from previously published studies conducted in low-income and middle-income countries, which reported a prevalence of 2.6%, as well as the SSA region, where the prevalence was noted at 2.0%. [25,26]. While the previous studies are important and commendable, it is essential to underscore that our study’s findings capture the current socioeconomic conditions, utilizing the most recent datasets available. Furthermore, the variation in the prevalence of tobacco product use across individual countries could also show the cultural influence and differences of tobacco product use across countries.

In this study, pregnant women who resided in high-wealth index households were less likely to use tobacco products compared to those who resided in low-wealth index households, consistent with previously published studies [27–29]. The reciprocal association between wealth and smoking could be explained by the possibility that pregnant women in poverty might resort to smoking as a form of self-medication to control their emotions, cope with stress, and ease the tensions associated with financial hardship [29,30]. Policy implications arising from our findings underscore the importance of targeted interventions and support for pregnant women in low-income households.

The results of this study revealed that pregnant women aged 25 years and above were associated with a higher risk of smoking, consistent with previous studies [31–33]. While smoking has been a long-standing cultural tradition in some communities, especially with smokeless tobacco products like snuff and snus, younger women may have more access to current health information sources, making them more aware of the risks associated with tobacco use [34,35]. Targeted interventions are imperative to address the unique challenges faced by the older age group, promoting healthier behaviors during pregnancy.

Pregnant women with primary, secondary, and higher education levels are less likely to use tobacco products than pregnant women without formal education, aligning with prior studies [36,37]. Our findings could be explained by three pathways. Firstly, education may increase awareness about the health risks of tobacco use, empowering women to make informed decisions for their health and that of their unborn child [38]. Secondly, educated women may have better access to resources, support networks, and healthcare services, creating an environment for healthier lifestyle choices [39]. Lastly, education can contribute to greater socioeconomic stability, reducing the chance of using smoking as a coping mechanism in times of stress [40]. Promoting universal primary education is crucial for fostering healthier behaviors among pregnant women and improving maternal and fetal health outcomes.

Furthermore, pregnant women exposed to media were less likely to use tobacco products, consistent with earlier studies [41]. Media plays a crucial role in influencing population smoking behaviors. Media campaigns that discourage tobacco use have the power to reshape people’s perceptions, prevent smoking initiation, and support adult quitting. Attitudes toward tobacco are significantly influenced by media communications, and evidence indicates that exposure to media messages about tobacco use impacts both its tobacco use and prevention [42,43]. Our findings suggest the importance of prioritizing the implementation of media campaigns to effectively convey anti-tobacco messages to pregnant women and the broader population.

## Limitations and strengths of the study

This study has limitations. Firstly, the cross-sectional nature of the data makes it challenging to establish causality. Secondly, the measurement of tobacco use relies on self-reporting, introducing the possibility of underreporting due to social desirability bias. Thirdly, the surveys were conducted at different times in each respective country, caution must be exercised when comparing the prevalence of tobacco use between nations. Despite the limitations, the study used nationally representative DHS data sets from 33 SSA countries, enhancing the generalizability of our findings. Additionally, the DHS uses standardized methods and instruments to ensure consistency in survey design and data collection.

## Conclusion and recommendation

This study provides timely evidence on the overall prevalence of tobacco use among pregnant women in SSA countries, using nationally representative data. While the overall prevalence is relatively low across 33 SSA countries, certain nations exhibit higher rates. Multilevel multivariable logistic regression results indicate that younger pregnant women, those with primary and secondary education, higher household wealth, and media exposure are less likely to use tobacco.

Educational interventions are crucial for women without formal education. Primary care settings are an essential source of prenatal care education for pregnant women from low-income households. Older women especially those who have no education and media exposure should be informed that smoking does not reduce stress levels. Enhancing awareness through education and offering smoke cessation programs through mass media and as part of antenatal care is crucial.

Despite a lower overall prevalence compared to high-income nations, specific SSA countries report high levels of tobacco use during pregnancy. This underscores the importance of targeted interventions in these countries to raise awareness about the health risks of tobacco use among women, especially during pregnancy.

## Supporting information

S1 File (PDF)

S2 File (PDF)
