# Supplementary material for: Determinants of tobacco use among pregnant women in sub-Saharan Africa. A multilevel mixed-effect logistic regression model
Source: PLoS One. 2024 May 21;19(5):e0297021. doi: 10.1371/journal.pone.0297021 (PMC11108210; doi:10.1371/journal.pone.0297021)
Supplement: S2 File — (PDF) [file pone.0297021.s002.pdf]

Gmail **Setognal Birara <geez4214@gmail.com>**

ACTION REQUIRED for PONE-D-23-18337

Abay Woday Tadesse <abaywoday@yahoo.com> Wed, Apr 10, 2024 at 10:10 PM
Reply-To: Abay Woday Tadesse <abaywoday@yahoo.com>
To: plosone <plosone@plos.org>
Cc: Setognal Birara <geez4214@gmail.com>

Dear Nikki Vea Orong,

I agreed with the amendment and attached the snapshot of my agreement below.

Best regards!

Abay W. Tadesse

On Wednesday, April 10, 2024 at 08:05:14 PM GMT+8, Abay Woday Tadesse <abaywoday@yahoo.com> wrote:

Dear team,

Thank you for contacting me!

Yes, I am co-author of this article and I agreed with the amendment.

Best regards!

Abay W. Tadesse
Alternative email: abay.tadesse@curtin.edu.au

On Saturday, February 17, 2024 at 01:02:10 AM GMT+8, plosone <plosone@plos.org> wrote:

[Quoted text hidden]

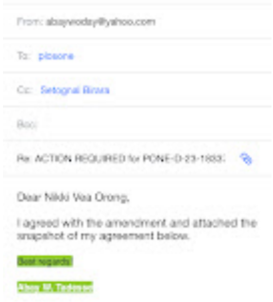
From: abaywoday@yahoo.com
To: plosone
Cc: Setognal Birara
Bcc:
Re: ACTION REQUIRED for PONE-D-23-1833[illegible]
Dear Nikki Vea Orong,
I agreed with the amendment and attached the snapshot of my agreement below.
Best regards
Abay W. Tadesse

IMG_4095.jpeg
345K

Gmail **Setognal Birara <geez4214@gmail.com>**

ACTION REQUIRED for PONE-D-23-18337

Abubeker Alebachew SEID <abubekeralebachew11@gmail.com> Sat, Feb 17, 2024 at 4:20 AM
To: plosone <plosone@plos.org>
Cc: Kusse Urmale <kussesinbo@gmail.com>, betelhem dagnew <betelhem.dagnew@yahoo.com>, mequsharew8@gmail.com, kebedegemeda2008@gmail.com, Abay Woday <abaywoday@yahoo.com>, kahmed@csu.edu.au, Setognal Birara <geez4214@gmail.com>

I agree with the proposed authors list.

Abubeker,
[Quoted text hidden]

Gmail

Setognal Birara <geez4214@gmail.com>

ACTION REQUIRED for PONE-D-23-18337

Samiha <betelhem.dagnew@yahoo.com> Sun, Feb 18, 2024 at 11:34 PM
Reply-To: Samiha <betelhem.dagnew@yahoo.com>
To: plosone@plos.org, "kussesinbo@gmail.com" <kussesinbo@gmail.com>, "abubekeralebachew11@gmail.com" <abubekeralebachew11@gmail.com>, "mequsharew8@gmail.com" <mequsharew8@gmail.com>, "kebedegemeda2008@gmail.com" <kebedegemeda2008@gmail.com>, "abaywoday@yahoo.com" <abaywoday@yahoo.com>, "kahmed@csu.edu.au" <kahmed@csu.edu.au>
Cc: "geez4214@gmail.com" <geez4214@gmail.com>

Hello dear PLOS Team, this is my confirmation to amend the author list on the PLOS ONE manuscript.

Best,
Betelhem.
Sent from Yahoo Mail on Android
[Quoted text hidden]

Gmail **Setognal Birara <geez4214@gmail.com>**

Approval of Amendment to Authors' List

Kebede Gemeda <kebedegemeda2008@gmail.com> Thu, Feb 29, 2024 at 5:40 PM
To: plosone@plos.org
Cc: Setognal Birara <geez4214@gmail.com>

Dear Journal Editorial Assistant,

I am writing in response to the request for each co-author to approve the amendment to the authors' list for our manuscript titled "Determinants of tobacco use among pregnant women in Sub-Saharan Africa: A multilevel mixed-effects logistic regression model" (PONE-D-23-18337). I hereby approve the requested amendment to the author list.

Thank you for your attention to this matter.

Warm regards,
Kebede Gemeda

Gmail

Setognal Birara <geez4214@gmail.com>

ACTION REQUIRED for PONE-D-23-18337

Ahmed, Kedir <kahmed@csu.edu.au> Sat, Feb 17, 2024 at 7:50 AM
To: plosone <plosone@plos.org>, "kussesinbo@gmail.com" <kussesinbo@gmail.com>, "betelhem.dagnew@yahoo.com" <betelhem.dagnew@yahoo.com>, "abubekeralebachew11@gmail.com" <abubekeralebachew11@gmail.com>, "mequsharew8@gmail.com" <mequsharew8@gmail.com>, "kebedegemeda2008@gmail.com" <kebedegemeda2008@gmail.com>, "abaywoday@yahoo.com" <abaywoday@yahoo.com>
Cc: "geez4214@gmail.com" <geez4214@gmail.com>

Dear PLOS One Team,

This a confirmation of my agreement to the proposed author list change.

Best regards,

Kedir

Dr Kedir Ahmed

Research Fellow of Rural Public Health (Epidemiologist)

Rural Health Research Institute

Charles Sturt University, Orange, NSW

Ph: 02 6365 7125

Email: kahmed@csu.edu.au

Publications: https://researchoutput.csu.edu.au/en/persons/kedir-ahmed

‘We pay our respect to the Wiradjuri, Ngunawal, Gundungarra and Birpai peoples of Australia, who are the traditional custodians of the land where Charles Sturt University campuses are located.’

[Quoted text hidden]

Gmail

Setognal Birara <geez4214@gmail.com>

ACTION REQUIRED for PONE-D-23-18337

Kusse Urmale <kussesinbo@gmail.com> Fri, Feb 16, 2024 at 10:44 PM
To: plosone <plosone@plos.org>
Cc: Setognal Birara <geez4214@gmail.com>

I confirm that the author list is correct.

[Quoted text hidden]

Gmail **Setognal Birara <geez4214@gmail.com>**

ACTION REQUIRED for PONE-D-23-18337

mequannent sharew <mequsharew8@gmail.com> Sun, Feb 18, 2024 at 11:27 PM
To: plosone <plosone@plos.org>
Cc: Kusse Urmale <kussesinbo@gmail.com>, betelhem.dagnew@yahoo.com, abubekeralebachew11@gmail.com, kebedegemeda2008@gmail.com, abay woday <abaywoday@yahoo.com>, kahmed@csu.edu.au, Setognal Birara <geez4214@gmail.com>

I agreed to the proposed author list change.

Mequannent

On Fri, Feb 16, 2024, 8:02 PM plosone <plosone@plos.org> wrote:
[Quoted text hidden]
